# A tentative guide for thoracic surgeons during COVID-19 pandemic

**DOI:** 10.1186/s43057-020-00026-z

**Published:** 2020-07-02

**Authors:** Ahmed Ghoniem, Amr Abdellateef, Amr Ibrahim Osman, Hany Hasan Elsayed, Hussein Elkhayat, Waleed Adel

**Affiliations:** 1 Cardiothoracic Surgery Department, Assiut University Hospitals, Assiut University, Assiut, 71526 Egypt; 2 Mansoura University, Mansoura, Egypt; 3 Ain Shams University, Cairo, Egypt; 4 Cairo University, Giza, Egypt

**Keywords:** COVID-19, Infection control, Thoracic, Surgery

Currently, there is not enough evidence to support the best practice for thoracic surgery practice during pandemic and the situation is highly dynamic on day to day basis.

Thoracic surgery may not seem to be in the frontline with managing COVID-19 patients, but we do have a key role to play and this must be planned. In response to pressures of the current pandemic, the elective component of our work has stopped. However, the non-elective patients (emergency, urgent, and trauma) will continue to need appropriate care and reactivation of service after situation becomes more stable.

We should seek to provide the best local solutions in conjunction with the recent national guidelines to continue the proper management of patients while ensuring proper resource allocation in response to COVID-19 with proper protection to the surgical stuff. In addition, we need to consider the possibility that the surgical facility for emergency surgery may be compromised in the near future due to a combination of factors including medical staff sickness, supply chain, and the use of theaters and anesthetic staff work in ICU instead. This is a possible scenario and plans are needed.

As the wider healthcare response intensifies, we may also need to work outside of our specific areas of training and expertise, and we must plan to learn new skills and practice.

Proper communication (could be telecommunication) with cardiothoracic patients awaiting operations is a must in the for-coming period. Explaining that there is no harm to wait for a few weeks for appropriate cases and the plan to allocate resources can provide re assurance for many elective patients feeling the danger of delay. Maximize virtual follow-up (phone call check) to avoid the need for patients to attend hospital. Consenting patients for surgery in this period should always include a hospital-acquired risk to develop a COVID-19 infection.

In this review, we try to point out the idea of triage of thoracic surgery patients, precautions for use of bronchoscopy, and chest tubes as the most procedures done with possible aerosol generation and when can we reactivate our thoracic surgery service.

## Thoracic surgery triage during pandemic

### Phase 1 (semi-urgent cases) of the COVID-19

Here, the hospital resources are not exhausted, COVID trajectory is not in a rapid escalation phase, and the tertiary center ICU vent capacity is still available. Surgical services are offered for those where survival could be affected in the next few days if no intervention is performed (Table 1) [1].

**Table 1:** Summary for decision-making in different pathologies during phase 1

Pathology	What to operate	What to defer
NSCLC	• Solid or predominantly solid (> 50%) lung cancer or presumed lung cancer ≥ 2 cm, clinical node negative • Post-induction therapy cancer	• Predominantly ground glass (< 50% solid) nodules or cancers • Solid nodule or lung cancer < 2 cm • Indolent histology (e.g., carcinoid, slowly enlarging nodule) ➢ Alternatives include: • SABR, neoadjuvant therapy • Ablation, stent, or debulking for endobronchial lesions
Esophageal tumors	• Esophageal cancer T1b or greater • Stenting for obstructing esophageal tumor	Esophageal cancer T1a/b (superficial) could be managed endoscopically.
Chest wall	Chest wall tumors of high malignant potential not manageable by alternative therapy	Chest wall tumors of high malignant potential manageable by alternative therapy
Mediastinal tumors	Symptomatic mediastinal tumors—diagnosis not amenable to needle biopsy	• Thymoma (non-bulky, asymptomatic) • Posterior mediastinal neurogenic tumors
Other oncothoracic interventions	Staging to start treatment (mediastinoscopy, diagnostic VATS for pleural dissemination)	Pulmonary oligometastases—unless clinically necessary for pressing therapeutic or diagnostic indications (i.e., surgery will impact treatment)
Others	• All emergency cases as massive hemothorax, major airway injury, airway obstruction by inhaled foreign body or advanced tracheal stenosis, and diaphragmatic hernia with strangulation • Loculated empyema with sepsis that cannot otherwise be treated • Tension emphysematous bullae with respiratory distress • Recurrent pneumothorax with massive air leak	• Pectus surgery • Hyperhidrosis • Bronchiectasis • Tracheal resection in tracheostomized patients • Non-malignant pleural effusion • Elective bullectomy

*SBRT* stereotactic body radiation therapy, *VATS* video-assisted thoracoscopic surgery

No standard definition exists so far for the urgent cases that need to be done nor consensus on which patient cannot wait without further progression and surgeons will always balance between patients with risk of significant deterioration and availability of resources [2].

Avoid the routine use of critical care unit wherever possible (pre-op preparation, ERAS, pain relief)

### Phase 2: urgent setting

In this phase we see many COVID-19 patients with limited ICU capacity and an escalating COVID trajectory.

Surgical management only offered for those who would not survive for few days if not operated upon (Table 2) [1].

**Table 2:** Summary for decision-making in different pathologies during phase 2

Pathology	What to operate	What to defer
NSCLC	• Tumor-associated infection—compromising, but not septic (e.g., debulking for post-obstructive pneumonia) • Tumor associated with hemorrhage, not amenable to nonsurgical treatment. • Threatened airway	• As phase 1 in addition to any non-complicated NSCLC by infection or hemorrhage or airway obstruction • Alternatives as phase 1 in addition to referral to phase 1 hospitals
Esophageal cancer	Septic or non-septic perforation only	Non-complicated by perforation cases
Postoperative complications (hemothorax, empyema, infected mesh, dehiscence of airway, anastomotic leak with sepsis)	Hemodynamic stable or unstable patients	Minor wound infections
Others	• All emergency cases as massive hemothorax, major airway injury, airway obstruction by inhaled foreign body or advanced tracheal stenosis, and diaphragmatic hernia with strangulation • Loculated empyema with sepsis that cannot otherwise be treated • Tension emphysematous bullae with respiratory distress • Recurrent pneumothorax with massive air leak	• Pectus surgery • Hyperhidrosis • Bronchiectasis • Tracheal resection in tracheostomized patients • Non-malignant pleural effusion • Elective bullectomy • Retained bullets with no fear of migration or embolization • Empyema that can be drained by chest tube • Pneumothorax for pleurodesis

*NSCLC* non-small cell lung cancer

### Phase 3: full outbreak of COVID 19

All resources are routed to COVID-19 patients and there is no ICU capacity.

Thoracic surgery procedures are only performed for patients who are likely to die within the next few hours if no intervention is performed (Table 3) [1].

**Table 3:** Summary for decision-making in different pathologies during phase 3

Pathology	What to operate	What to defer
NSCLC	• Threatened airway • Tumor-associated severe infection • Hemodynamically unstable patients with tumor-associated hemorrhage	As phase 1, 2 plus hemodynamically stable patients with any complication
Esophageal cancer	Septic perforation	As phase 1, 2 plus non-septic perforation
Postoperative complications	Only hemodynamically unstable patients, critically compromised airway as active bleeding not amenable to nonsurgical management, dehiscence of airway, and anastomotic leak with sepsis.	As phase 2 plus any complication not critically compromising the airway or causing hemodynamic instability
Trauma	All emergency cases as massive hemothorax, major airway injury, airway obstruction by inhaled foreign body or advanced tracheal stenosis, and diaphragmatic hernia with strangulation	As phase 2 plus any complication not critically compromising the airway or causing hemodynamic instability

### Trauma patients

Management of trauma patients is highly dependent on healthcare facility because even if COVID-19 screening test is available at initial assessment, negative test results do not exclude the presence of infection [2].

So, if the patient needs emergency interventions, he should be considered as a positive case with the following precautions:

- Full personal protective equipment (gowns, gloves, surgical masks, and face shield) are highly recommended.
- Minimize transfer these patients to multiple areas inside the hospital.
- Diagnostic procedures that would not affect the decision should be avoided.
- All members of the trauma unit should be familiar with the infection control protocols.

If the patient needs urgent or elective interventions, CBC, body temperature, and CT chest should be done first and reported if consistent with COVID-19 or not.

## Reactivation of thoracic surgery service

Returning to the usual surgical routine is within the scope of every surgeon now. Too many factors need to be taken in consideration; the limited resources as chain of supplies, number of nurses, available ICU beds, personal protective equipment (PPE), the limited access to diagnostic procedures as pulmonary function tests, bronchoscopy and the availability of PCR, and other tests for every patients and medical stuff. Early results from CovidSurg study show that patients whom discovered to be COVID positive perioperatively had a high complication and mortality rates [3], making the decision for operating every non-urgent case during the pandemic without rolling out the possibility of infection is a high risk operation. As a result, a very large number of operations will be postponed due to disruption caused by COVID-19 and in a recent study they concluded that it would take a median 45 weeks to clear the backlog of operations resulting from COVID-19 disruption if surgery services increase the normal surgical volume rate by 20% during the post-pandemic era [4].

### Precautions for bronchoscopy

- Bronchoscopic procedures are aerosol-generating procedures (AGPs).
- Indications for bronchoscopy should consider the potential for transmission of COVID-19 infection.
- All foreign body inhalation patients with respiratory distress should have their bronchoscopy done without delay.
- Make sure that your bronchoscopy set is sterilized (autoclave preferred) before starting the procedure
- Minimize the personnel in OR to the least possible
- Standard Personal protective equipment (surgical masks, eye protection) for all bronchoscopy patients
- Avoid high flow jet oxygen ventilation whenever possible

For cases of suspected COVID-19, this should only occur in exceptional circumstances when bronchoscopy cannot be deferred and (FFP3 respirator, long-sleeved gown, gloves, eye protection) should be worn and safely taken off and dispose appropriately as instructed by infection control staff.

### Chest tube in era of COVID-19

#### Indications and triage

Insertion of intercostal drain (ICD) during COVID-19 pandemic could be indicated for a well-known hospitalized COVID-19-positive patients or usual patients who visit any hospital emergency department for traumatic or non-traumatic pneumothorax or pleural effusion. Despite presence of theoretical higher risk of infection in case of dealing with COVID-19-positive patient, healthcare workers’ (HCW) precautions may minimize that risk as HCW would take complete caution by wearing personal protective equipment (PPE) and by keeping vigilant not to catch infection. Actually, the silent risk is to go for ICD insertion in a COVID-19 asymptomatic carrier [5].

The symptoms that carry the indication for chest tube insertion could mask the symptoms of a COVID-19 infection. Examples include empyema and large pneumothoraces causing fever and dyspnea, similar to COVID-19 symptoms, so a triage can be carried out primarily and further categorization of patients according to urgency is better to be based on special protocol or algorithm for every thoracic surgery unit. Any patient should be dealt with as assumed COVID positive until proved otherwise [6, 7].

### Preparation

#### Preparation of drainage system

Higher risk of aerosol production is raised in cases of pneumothorax with active air leak. The most common available drainage system at our community is the traditional under water-seal collectors which have an outlet vent to atmosphere. When air passes from thoracic cavity to the water in the containers, it causes bubbling with the transmission of air via the outlet vent to the atmosphere causing potential environmental aerosol viral infection. Also, even though digital chest drainage systems do not have an outlet vent to room air, they are not closed systems and the air may escape from them into the air without any specific viral filter [8].

So, in spite of closed suction drainage system is advised to be used to limit aerosolization, there is a general acceptance on use of viral filter as purifying media between the drain and the suction system or the atmosphere whatever the open or closed drainage system is used. Site of application of the viral filter would be distal to the outlet vent. According to the recently published reports, different forms of high-efficiency particulate air (HEPA) viral filters have been used [6–8].

Use of viral filter in chest tubes has not been examined yet on evidence-based criteria, but it depends on the rationale of its proved ability to filter smaller viruses like hepatitis C whose average diameter is of about 55 nm compared to SARS Cov-2 diameter which varies from 60 to 140 nm [8].

Lessening of viral load also could be managed at an earlier level through adding sterilizing solution like; dilute household bleach (5.25–6.15% sodium hypochlorite) with ratio of 1:50 to the fluid in the water seal [6] ordinary betadine used for wound care and alcohol 70% were also used by many senior surgeons as they used to do in the TB era.

**Fig. 1:**
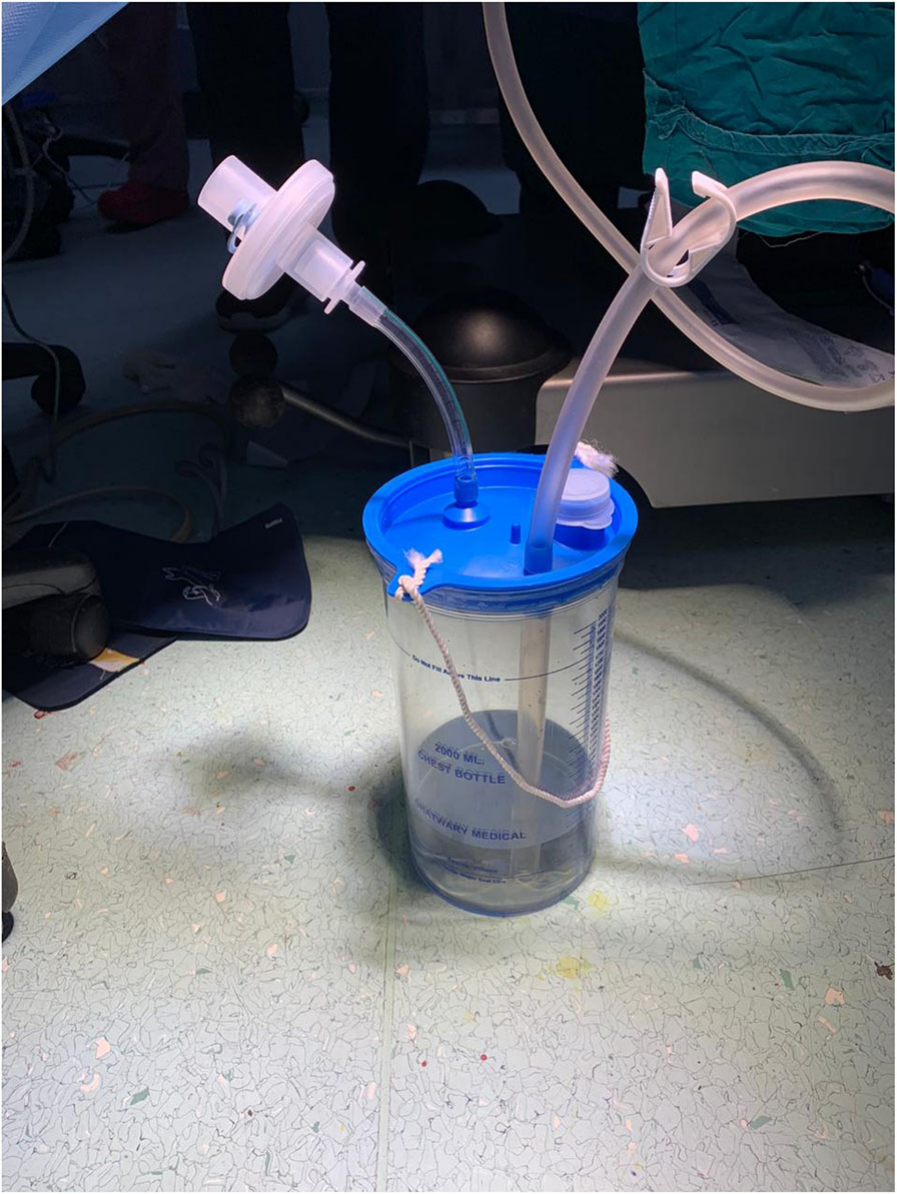
Preparation of the drainage system

In advance preparation of the drainage system depends on creation of ready sterile connected set starts up by a suitable size chest drain firmly connected through a connector to an underwater seal container having sterile solution and its outlet vent is connected to HEPA filter through a cut endotracheal tube (Fig. 1). This pre-prepared drainage system is so crucial to prevent any potential aerosol infection in the time consumed preparing the drainage system after insertion of the ICD inside the thoracic cavity.

#### Precaution during the technique

-Before starting the procedure, prepare the whole connected drainage set including the drain and underwater seal carrying a viral filter as described before.

-It is better to direct the patient’s face to the opposite side during the procedure and advise him to wear a surgical mask to reduce viral load exhaled (if not impairing his breathing).

-Make the skin incision as small as possible to avoid air or fluid leak around the tube.

-Once you open the pleura, take care of the first gush of air due to the sudden decompression that carry the potential maximum viral load. To lessen that gush, the surgeon can cover the skin incision by a wet gauze with the non-working hand while the other hand at the same time opening the pleura or inserting the tube. That gauze works as a valve mechanism to prevent exit of air through the skin till insertion of the tube.

-The maximum air leak and bubbling will be at the start with maximum viral load getting out through the vent passing by the viral filter. So, the underwater seal drain could be put temporarily in a loosely closed plastic bag to contain any increased aerosolization after primary pleural decompression, then that plastic bag would be removed.

-If the patient is mechanically ventilated, hold ventilation before entry into the pleural space or until connection of the tube to the drain if not.

-Tighten the skin incision around the tube to prevent any air leakage.

-After finishing the procedure, get rid of disposables along with PPE as per recommended local institutional protocol.

#### Follow-up of the drain

-Usual follow-up or dressing on the wound should be conducted by HCW while wearing N95 mask, face shield, eye google, gloves, and non-sterile gown.

-Testing of residual air leakage by asking the patient to cough or to take deep breath should be minimized. If it is necessary in case of absent digital calculation system of air leak, the patient should wear a mask and look at the opposite direction while doing that.

-Do not overhandle the tube, e.g., do not pull the tube if kinked as long as it is functioning well. Pulling out the tube will take the contaminated intrathoracic part exposed out.

-If needed, clamp the tube while changing the bottle system or connecting it.

-Follow-up with radiology is better to be done by portable x-ray device inside the ward or inside a specific radiology unit for COVID-positive patients with predetermined transport routes to decrease the possible hospital environmental contamination [9].

#### Removal of the drain

-Removal of the drain should be in a specific dressing room while wearing full PPE.

-Upon removal of the tube, the procedure should be airtight to prevent any air to get in or out from chest cavity. That could be done through pinching of the skin around the skin opening or covering the skin by a gauze soaked by ointment while pulling out the tube followed by fast tightening of the preplaced suture [6].

## Data Availability

Not applicable

## Abbreviations

AGP: Aerosol-generating procedures
ERAS: Enhanced recovery after surgery
FFP3: Filtering facepiece class 3
ICU: Intensive care unit
NSCLC: Non-small cell lung cancer
OR: Operating room
SBRT: Stereotactic body radiation therapy
VATS: Video-assisted thoracoscopic surgery

## References

[1] Thoracic Surgery Outcomes Research Network I (2020) COVID-19 Guidance for Triage of Operations for Thoracic Malignancies: A Consensus Statement from Thoracic Surgery Outcomes Research Network. Ann Thorac Surg S0003-4975(20):30442–2. 10.1016/j.athoracsur.2020.03.005 10.1016/j.athoracsur.2020.03.005 PMC7146713 32278755

[2] Brian, M. and W. Douglas E. 2020, Definition of necessary surgery in the age of COVID-19: an interview with Douglas E. Wood.

[3] Collaborative CO (2020) Mortality and pulmonary complications in patients undergoing surgery with perioperative SARS-CoV-2 infection: an international cohort study. Lancet 10.1016/S0140-6736(20)31182-X PMC7259900 32479829

[4] COVIDSurg Collaborative, D. Nepogodiev, and A. Bhangu (2020) Elective surgery cancellations due to the COVID ‐19 pandemic: global predictive modelling to inform surgical recovery plans. Br J Surg. 10.1002/bjs.11746 10.1002/bjs.11746 PMC7272903 32395848

[5] Li R et al (2020) Substantial undocumented infection facilitates the rapid dissemination of novel coronavirus (SARS-CoV-2). Science 368(6490):489–493 32179701 10.1126/science.abb3221 PMC7164387

[6] Pieracci FM et al (2020) Tube thoracostomy during the COVID-19 pandemic: guidance and recommendations from the AAST Acute Care Surgery and Critical Care Committees. Trauma Surg Acute Care Open 5(1):e000498 32411822 10.1136/tsaco-2020-000498 PMC7213907

[7] CARVALHO EDA, OLIVEIRA MVBD (2020) Safety model for chest drainage in pandemic by COVID-19. Revista do Colégio Brasileiro de Cirurgiões 47 10.1590/0100-6991e-20202568 32490892

[8] Rajdeep, B., et al., COVID-19: chest drains with air leak – the silent ‘super spreader’? 2020.

[9] World Health Organization. 2020 Infection prevention and control during health care when novel coronavirus (nCoV) infection is suspected [cited 2020 5 june 2020 ]; Available from: https://www.who.int/publications/i/item/infection-prevention-and-control-during-health-care-when-novel-coronavirus-(ncov)-infection-is-suspected-20200125.

